# Same Song, Different Audience: Pharmaceutical Promotion Targeting Non-Physician Health Care Providers

**DOI:** 10.1371/journal.pmed.1001560

**Published:** 2013-11-26

**Authors:** James S. Yeh, Aaron S. Kesselheim

**Affiliations:** Program On Regulation, Therapeutics, And Law (PORTAL), Division of Pharmacoepidemiology and Pharmacoeconomics, Department of Medicine, Brigham and Women's Hospital and Harvard Medical School, Boston, Massachusetts, United States of America

**Linked Research Article**

This Perspective discusses the following new study published in *PLOS Medicine*:

Grundy Q, Bero L, Malone R (2013) Interactions between non-physician clinicians and industry: A systematic review. PLoS Med (10)11: e1001561. doi:10.1371/journal.pmed.1001561

In a systematic review of studies of interactions between non-physician clinicians and industry, Quinn Grundy and colleagues found that many of the issues identified for physicians' industry interactions exist for non-physician clinicians.

Promotion of medical products by manufacturers to physicians in the US has been widespread for over a century [1], and in its traditional form includes strategies such as direct detailing visits (in-person product promotion by a pharmaceutical representative), sponsorship of Continuing Medical Education programs, gifts or other inducements, and free samples [2]. In the past two decades, numerous studies have shown that these interactions shape clinical knowledge and medication-prescribing behavior in ways that are not consistent with evidence-based practices [3],[4]. As a result, some physicians and policymakers have sought to mitigate their influence. For example, many hospitals and academic medical centers have established policies that require disclosure of certain behaviors that create conflicts of interest—like service on a manufacturer's speaker's bureau—or limit excessive non-research consulting payments that physicians may receive [5]–[7]. These local efforts have now been supplemented in the US by the Centers for Medicare and Medicaid Services' Open Payments program (created by the Physician Payments Sunshine Act) [8], which began receiving comprehensive information from drug and medical device manufacturers in August 2013 about all payments made to physicians and hospitals and plans to publicly disseminate the data starting in 2014.

With greater regulatory attention to traditional promotional practices, “non-traditional” forms of promotion remain an option for pharmaceutical and medical device companies. Non-traditional promotion can include scientific pursuits or other business relationships that might appear to be outside the realm of promotion but that are conducted for a marketing purpose, such as seeding trials or funding of patient advocacy organizations [9],[10]. It also includes targeting promotion to non-physician health care providers, such as nurse practitioners, pharmacists, and physician assistants. With recent changes in health care financing, these non-physician providers are taking an increasingly central role in care delivery, as medicine emphasizes interdisciplinary team-based care and integrative medical homes [11]. Thus, these providers offer a high-impact marketing alternative to traditional physician-directed promotion [12].

Grundy and colleagues, in this week's issue of *PLOS Medicine*, provide a systematic review of literature addressing the nature of non-physician providers' interactions with the medical products industry [13]. The authors identified fifteen studies published between 2003 and 2010 that investigated the type and frequency of interactions, non-physician providers' attitudes towards industry interactions, and the perceived effect of industry influence. The majority of the studies (11 out of 15) were conducted in the US, with participant size ranging from 14 to 1,640. One conclusion of their review was that nearly all of the US-based nurse practitioners responding in the studies have regular pharmaceutical representative contact (96%), have received industry-sponsored meals within the past 6 months (64%), have attended an educational event during the past five years paid for by the industry (96%), and have received free drug samples (66–86%).

The pervasive contact between non-physician providers and the medical products industry recalls a survey by Campbell and colleagues in 2004 that found 94% of physicians reporting recent interactions with pharmaceutical industry sales representatives [14]. Notably, a follow-up survey in 2009 found somewhat fewer physicians (84%) reporting such interactions, while a review of Massachusetts data from 2011 and 2012 suggests that only approximately one in four physicians received high-value monetary gifts (>$50) [15],[16]. Though these are not longitudinal data, they suggest that recent policy changes have been successful in insulating more physicians from promotional interactions. By contrast, the study from Grundy and colleagues shows that promotion to non-physician health care providers remains vibrant.

Current programs intended to provide some transparency about the prevalence of pharmaceutical industry marketing may not reach the non-physician provider population; most notably, the Open Payments program only gathers data on payments provided to physicians, not nurses, pharmacists, or other non-physician providers. Though this legislation could be revised to cover all providers of health care who are currently omitted from this federal mandate, we believe it is unlikely that Congress would be interested in revisiting the wording of this statute in the near future. Better regulation should be taken up at the local level, where institutional policies should be constructed to apply to all providers of patient care. For example, Partners HealthCare, the enterprise that manages our academic medical center, has implemented an industry interaction policy that applies to all employees [17].

In addition to the pervasiveness of industry interactions among non-physician providers, Grundy and colleagues also highlight parallels in how physicians and non-physician providers regard these interactions. For example, the review revealed that non-physician providers were confident that they themselves were immune to marketing influences and that they were able to detect biases while their own colleagues were less able to do so. This cognitive dissonance mirrors similar results found in physicians and physician trainees [18]. Grundy and colleagues also found that study participants generally have positive attitudes toward such industry interactions and did not support the need for policies to regulate their behavior. Thus, the review from Grundy and colleagues points to the persistent need for greater education among non-physician health care providers about how industry promotion tends to overestimate the benefits and underemphasize the risks of products.

Substantial progress has been made in recent years in raising awareness among physicians about the conflicts of interest that can arise in health care delivery and the effect of marketing on medical practices. It is time to shine a similar light on non-traditional forms of promotion, including marketing to non-physician providers.
